# Serotonin signalling in cancer: Emerging mechanisms and therapeutic opportunities

**DOI:** 10.1002/ctm2.1750

**Published:** 2024-06-28

**Authors:** Lulu Chen, Shuting Huang, Xiaoxue Wu, Weiling He, Mei Song

**Affiliations:** ^1^ Department of Gastrointestinal Surgery The First Affiliated Hospital of Sun Yat‐Sen University Sun Yat‐Sen University Guangzhou China; ^2^ Institute of Precision Medicine The First Affiliated Hospital of Sun Yat‐Sen University Sun Yat‐Sen University Guangzhou China; ^3^ School of Public Health Sun Yat‐Sen University Guangzhou China; ^4^ Department of Gastrointestinal Surgery Xiang'an Hospital of Xiamen University School of Medicine Xiamen University Xiamen China

**Keywords:** immunomodulatory function, serotonergic drugs, serotonin, serotonylation, tumourigenesis

## Abstract

**Background:**

Serotonin (5‐hydroxytryptamine) is a multifunctional bioamine serving as a neurotransmitter, peripheral hormone and mitogen in the vertebrate system. It has pleiotropic activities in central nervous system and gastrointestinal function via an orchestrated action of serotonergic elements, particularly serotonin receptor‐mediated signalling cascades. The mitogenic properties of serotonin have garnered recognition for years and have been exploited for repurposing serotonergic‐targeted drugs in cancer therapy. However, emerging conflicting findings necessitate a more comprehensive elucidation of serotonin's role in cancer pathogenesis.

**Main body and conclusion:**

Here, we provide an overview of the biosynthesis, metabolism and action modes of serotonin. We summarise our current knowledge regarding the effects of the peripheral serotonergic system on tumourigenesis, with a specific emphasis on its immunomodulatory activities in human cancers. We also discuss the dual roles of serotonin in tumour pathogenesis and elucidate the potential of serotonergic drugs, some of which display favourable safety profiles and impressive efficacy in clinical trials, as a promising avenue in cancer treatment.

**Key points:**

- Primary synthesis and metabolic routes of peripheral 5‐hydroxytryptamine in the gastrointestinal tract.
- Advanced research has established a strong association between the serotonergic components and carcinogenic mechanisms.
- The interplay between serotonergic signalling and the immune system within the tumour microenvironment orchestrates antitumour immune responses.
- Serotonergic‐targeted drugs offer valuable clinical options for cancer therapy.

## Graphical abstract

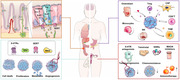

## 1 INTRODUCTION

Serotonin, also known as 5‐hydroxytryptamine (5‐HT), was initially named enteramine following its discovery in 1937 by Italian pharmacologist Vittorio Erspamer, who extracted it from enterochromaffin (EC) cells in the gastrointestinal (GI) tract.^1^ About a decade later (1948), Maurice Rapport and Irvine Page isolated a substance from bovine serum and called it *serotonin*, derived from the Latin term ‘serum’ (where it was discovered) and the Greek word ‘tonic’ (referring to its earliest known function).^2^, ^3^, ^4^ Enteramine was found to induce smooth muscle contraction and was considered as an exclusive signalling molecule in the GI tract until 1952, when Dr. Erspamer confirmed that enteramine and serotonin shared the same structure.^5^

The discovery of serotonin's noted function as a neurotransmitter can be traced back to 1953 in hard‐shell clams, which led to the subsequent identification of serotonergic system in the vertebrate brain.^6^, ^7^, ^8^ Its most noteworthy clinical role in the central nervous system (CNS) revolves around its function in psychiatric disorders such as depression, schizophrenia and anxiety. Thus, numerous pharmaceutical drugs were developed to target the serotonergic system, including antidepressants and antipsychotics.^9^ While the name *serotonin* stuck, the historical importance of enteramine has been recognised as the multifaceted GI roles of 5‐HT continue to be revealed. It acts as a peripheral hormone that plays a multitude of functions such as GI motility and emesis, vasoconstriction,^4^ angiogenesis,^10^ osteoporosis,^11^ wound healing and maintaining the glucose homeostasis and obesity.^12^ It also functions as a mitogen, modulating the cell cycle, inflammation and immunity. More recently, multiple studies have envisioned serotonin's carcinogenic properties, which sparked further investigation into its potential as a biomarker for carcinogenesis, the feasibility of serotonin pathways as potential therapeutic targets, and the repurposing of serotonergic‐targeted drugs (such as 5‐HT receptors [5‐HTRs] antagonists, serotonin reuptake inhibitors, serotonin synthesis inhibitors and monoamine oxidase inhibitors [MAOIs]) for cancer therapy. However, the functions of serotonin in cancer pathogenesis remain scanty and contradictory, largely attributed to the diversity and tissue‐specific distribution of 5‐HTRs and the complexity of serotonergic signalling. Here, we provide a holistic view of the serotonergic system and the signalling events downstream of serotonin. We then systematically discuss the original and intriguing signalling mechanisms of peripheral serotonin on tumourigenesis and summarise the latest advances in serotonergic‐targeted cancer therapies.

## 2 BIOSYNTHESIS AND METABOLISM OF 5‐HT

### 2.1 Biosynthesis of 5‐HT

**FIGURE 1:**
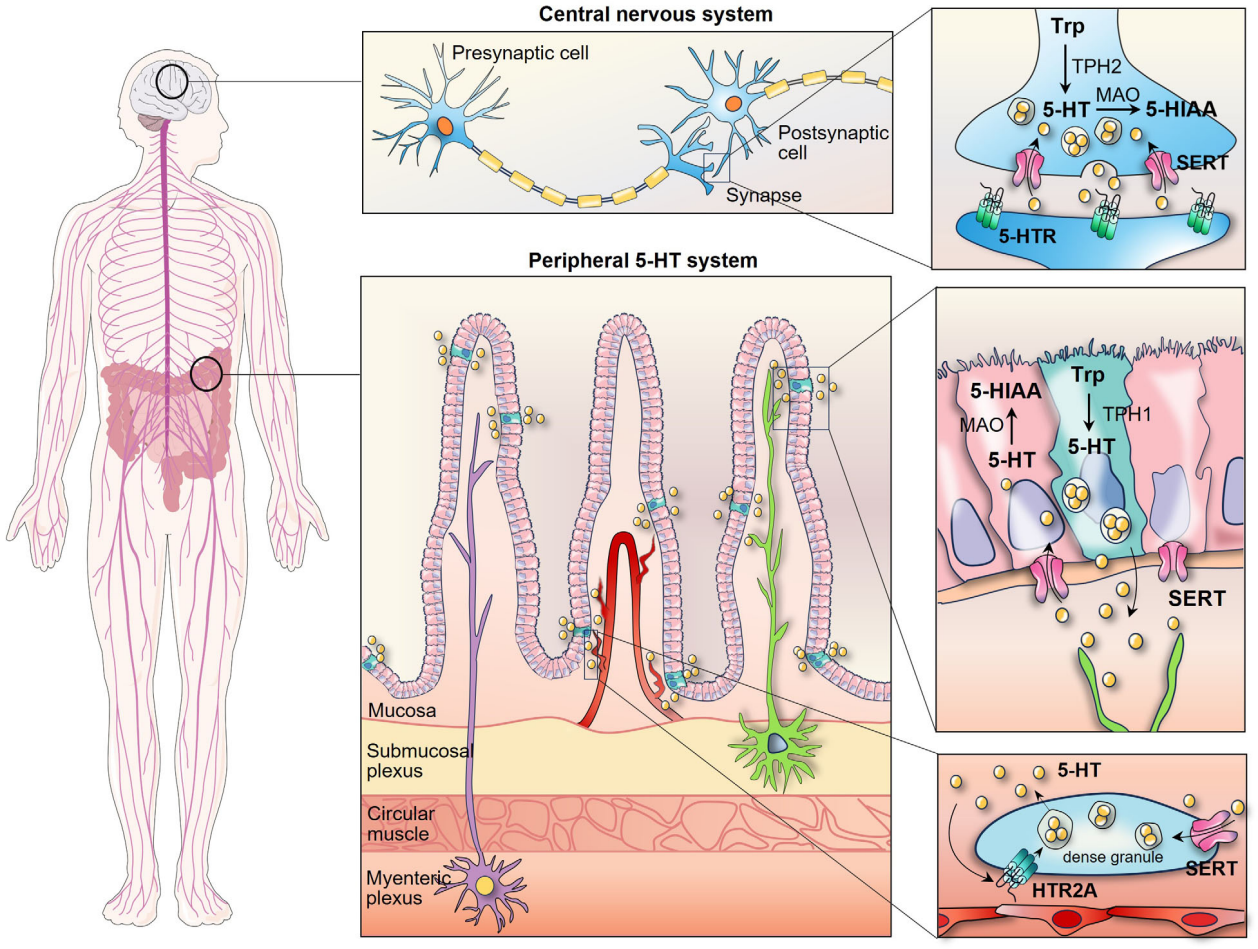
Schematic diagram of the life cycle of serotonin. Serotonin is synthesised from tryptophan in enterochromaffin (EC) cells and the central nervous system (CNS) by different isoforms of tryptophan hydroxylase (TPH). Serotonin derived from TPH1 in the mucosa enters the portal circulation and is then taken up by platelets via serotonin transporter (SERT). In the CNS, serotonergic neurons produce TPH2 to synthesise 5‐hydroxytryptamine (5‐HT), which is stored in synaptic vesicles. Once release from intracellular vesicles or platelets, 5‐HT binds to 5‐hydroxytryptamine receptors (5‐HTRs) on the cell membrane. The activation of 5‐HTR2A by 5‐HT can enhance the release of dense granules. The reuptake of 5‐HT is facilitated by SERT, which relocates the molecule intracellularly. Afterwards, monoamine oxidase (MAO) degrades 5‐HT into 5‐hydroxyindoleacetic acid (5‐HIAA) for inactivation. This figure was created using Adobe Illustrator and PowerPoint. Trp, tryptophan.

5‐HT is synthesised in the body through two steps involving the conversion of the dietary amino acid L‐tryptophan. The initial step is the rate‐limiting step and is catalysed by tryptophan hydroxylase (TPH). The second step involves decarboxylation of 5‐hydroxytryptophan through the enzymatic action of aromatic amino acid decarboxylase, as depicted in Figure 1. TPH exhibits two different isoforms: TPH2 is restricted to the CNS and enteric neurons, whereas TPH1 is widely distributed in the periphery and pineal gland, indicating the presence of two separate serotonin reservoirs in the body.^13^, ^14^ 5‐HT is produced and stored in presynaptic neurons within the CNS, where it primarily acts as a neurotransmitter. However, roughly 95% of the body's 5‐HT is believed to be synthesised by EC cells in the intestinal mucosa.^15^, ^16^ Interestingly, the production of 5‐HT in EC cells could be modulated by intestinal microbiota, which in turn, could be altered by 5‐HT.^17^, ^18^ Tumour‐associated microbiota also promotes 5‐HT production by augmenting *Tph2* expression.^19^ The intricate interplay between the microbiome and the serotonergic system is considered to regulate systemic serotonin homeostasis.

### 2.2 Storage and metabolism of 5‐HT

The majority of 5‐HT synthesised peripherally by EC cells is absorbed by platelets after being released into blood plasma via a serotonin reuptake transporter (SERT/SLC6A4), with less than 1% of serotonin circulating in the blood in an unbound state.^20^, ^21^ Platelets, as the main circulating reservoir of serotonin, have little ability to produce serotonin.^22^, ^23^ Generally, the serum serotonin level is typically maintained within the range of 1−5 ng/mL, but it can increase up to 1000‐fold when released from platelet dense granules at sites of inflammation or injury.^24^, ^25^ Intracellular serotonin sequestration facilitated by the vesicular monoamine transporter shields it from monoamine oxidase (MAO)‐mediated enzymatic degradation.^26^ MAO metabolises 5‐HT into 5‐hydroxyindoleacetic acid, that is, primarily eliminated through urine. The GI tract, brain and platelets are major sites where MAO activity exists. In addition, serotonin undergoes minor metabolic pathways such as glucuronidation and sulphation, which takes place in the liver, lung, kidney and brain.^27^ Within the CNS, MAO‐mediated metabolism occurs in the cytosol of the neuron, whereas in the pineal gland, serotonin is converted into melatonin through an alternative pathway. Due to its inability to penetrate the blood‒brain barrier, the central and peripheral serotonin systems are anatomically and functionally separate.^28^

## 3 FUNCTIONAL MODES OF SEROTONIN

### 3.1 Serotonin receptor families

**FIGURE 2:**
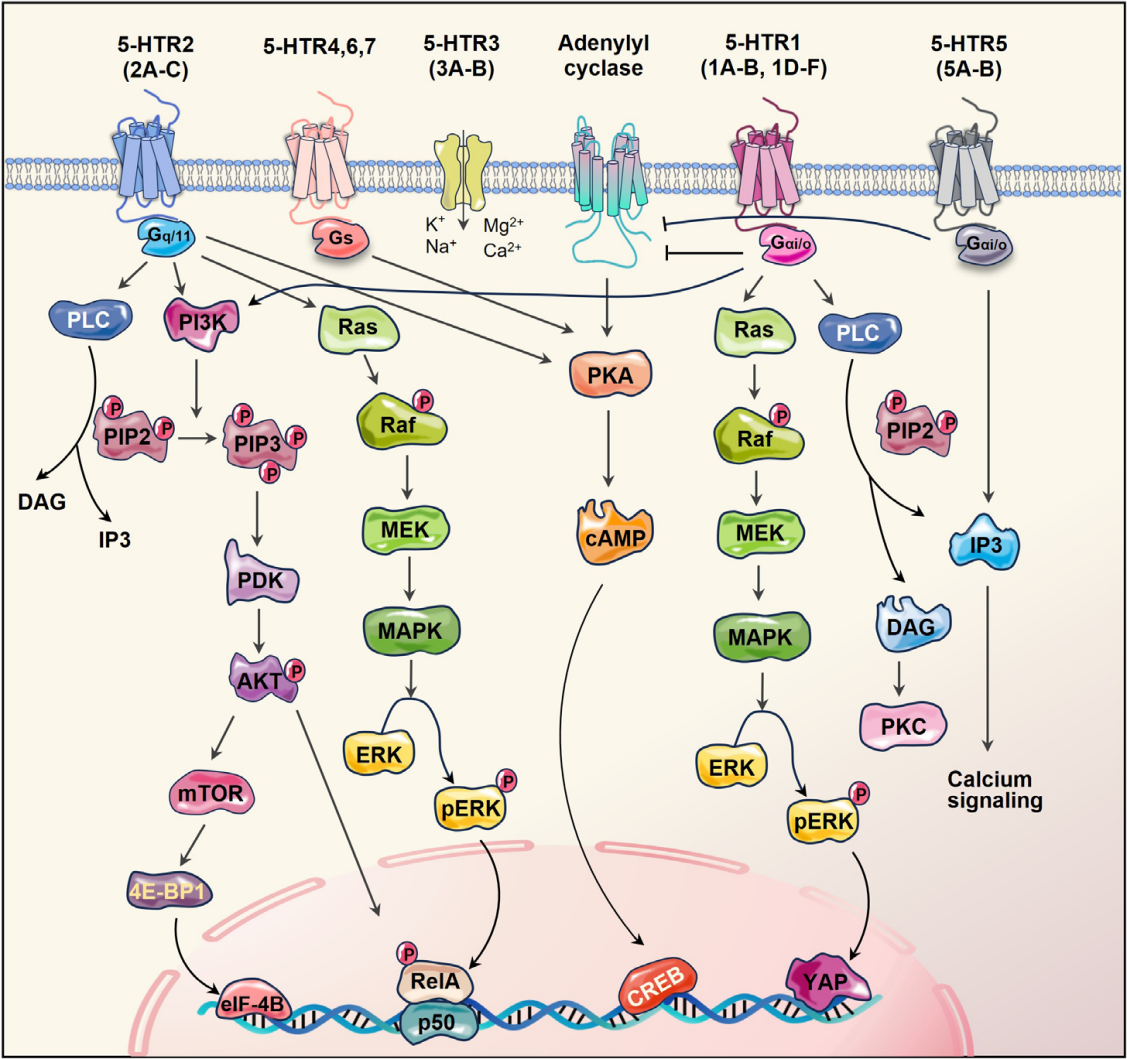
Serotonin receptor (5‐HTR) subtypes and signalling pathways. All 5‐HTRs are G‐protein‐coupled receptors, except for the 5‐HT3 receptor, which functions as a ligand‐gated channel. The seven distinct families of 5‐HTRs active four major interconnected signalling pathways: PI3K/AKT, PLC/DAG/PKC, AC‐PKA‐cAMP and Ras/Raf/MAPK/ERK axis. This figure was created using PowerPoint. AC, adenylate cyclase; AKT, protein kinase B; cAMP, cyclic adenosine monophosphate; CREB, cAMP‐response element binding protein; DAG, diacylglycerol; eIF‐4B, eukaryotic translation initiation factor 4B; ERK, extracellular signal‐regulated kinase; IP3, inositol triphosphate; MAPK, mitogen‐activated protein kinase; MEK, mitogen‐activated protein kinase; mTOR, mammalian target of rapamycin; PDK, phosphoinositide‐dependent kinase; PI3K, phosphoinositide 3‐kinase; PIP2, phosphatidylinositol (4,5) bisphosphate; PKA, protein kinase A; PKC, protein kinase C; PLC, phospholipase C; ReIA, v‐rel avian reticuloendotheliosis viral oncogene homologue A; 4E‐BP1, eukaryotic translation initiation factor 4E (eIF4E)‐binding protein 1; YAP, yes‐associated protein.

Our understanding of serotonin's function has significantly broadened over the last 20 years with the cloning of over 15 serotonin receptors, categorised into seven families based on the genetics and signalling mechanisms.^29^ Most receptors exhibited heterogeneity and were further subclassified. Of the families, 5‐HTR3 is distinctive since it engages a ligand‐gated Na^+^/K^+^ ion channel, while the rest six subtypes belong to the G‐protein‐coupled receptors.^30^ Generally, the 5‐HTR1 (1A‐1F) and 5‐HTR5 (5A/5B) families couple via inhibitory Gαi/o proteins and suppress adenylyl cyclase, resulting in the reduction of cyclic adenosine monophosphate (cAMP) levels. HTR1A exhibits cell type‐specific variations in its signalling repertoire, including extracellular signal‐regulated kinase 1/2 (ERK1/2), phosphoinositide 3‐kinase/protein kinase B (PI3K/AKT) and phospholipase C/protein kinase C (PLC/PKC). Whereas 5‐HTR4/6/7 activate adenylyl cyclase, increasing cAMP activity. In addition, 5‐HTR2 stimulates intracellular calcium signalling by activating PLC.^31^, ^32^ The distribution and subcellular location of diverse 5‐HTRs are crucial for determining the specific paracrine effects of 5‐HT. The complexity of serotonergic signalling is manifested by the activity of various 5‐HTRs that integrate multiple inputs into converging or diverging signals, ultimately resulting in a wide spectrum of physiological effects (Figure 2).

### 3.2 Serotonin transporter

The effects of 5‐HT rely on its availability, which is determined in part by the SERT. SERT uses sodium to transport monoamine and is accountable for clearing free serotonin from the extracellular space, thereby terminating the subseuent impacts of 5‐HTR activation. In the CNS, the depolarisation of neurons leads to the release of serotonin into the synaptic cleft, where it attaches to either postsynaptic 5‐HTR or presynaptic SERT.^33^ The interaction with SERT functions as a negative feedback mechanism, restraining additional serotonin discharge into the synaptic cleft.

Thus, the selective serotonin reuptake inhibitors (SSRIs), which specifically target SERT, increase the availability of serotonin at the synaptic junction, influencing the duration and intensity of 5‐HT signalling. These are the most widely used medications to treat obsessive compulsive disorder, depression and anxiety disorders.^34^ Treatment with SSRIs also leads to a depletion of platelet 5‐HT storage.

### 3.3 Receptor‐independent 5‐HT signalling—serotonylation

Serotonylation is a chemical modification through which 5‐HT is incorporated into acceptor proteins through the formation of glutamyl‐amide bonds in a transglutaminase (TGM)‐dependent way.^23^

Seven TGMs have been identified, primarily localising intracellularly, with TGM2 being the most ubiquitous and abundant one. Blood coagulation factor XIII, activated by thrombin during coagulation to form factor XIIIa, also exhibits extracellular TGM activity.^35^ Serotonylation occurs in both extracellular and intracellular compartments during thrombus formation, exemplifying this process in the body. Extracellularly, serotonylated procoagulant proteins bind to fibrinogen and thrombospondin, increasing the stability of essential protein complexes.^36^ Concurrently, 5‐HT attaches to platelet HTR2A, triggering the phosphatidylinositol pathway through a G_αq_‐protein‐dependent mechanism. This activation leads to a rise in cytoplasmic Ca^2+^, which is necessary for TGM activity.^23^ Once 5‐HT is transported into the cytoplasm, TGM crosslinks it to small G‐proteins (such as RhoA and Rab4), which constantly trigger α‐granule exocytosis.^23^ Due to the hydrophilic properties of 5‐HT, serotonylation is thought to exclusively occur in SERT‐expressing cells, including smooth muscle cells, pancreatic β cells, valve interstitial cells, neurons and glial cells.^37^ Besides platelet activation, serotonylation participates in various physiological functions, such as smooth muscle contraction,^38^ insulin release,^39^ dendritic spine plasticity^40^ and cardiac valve degeneration.^41^

**FIGURE 3:**
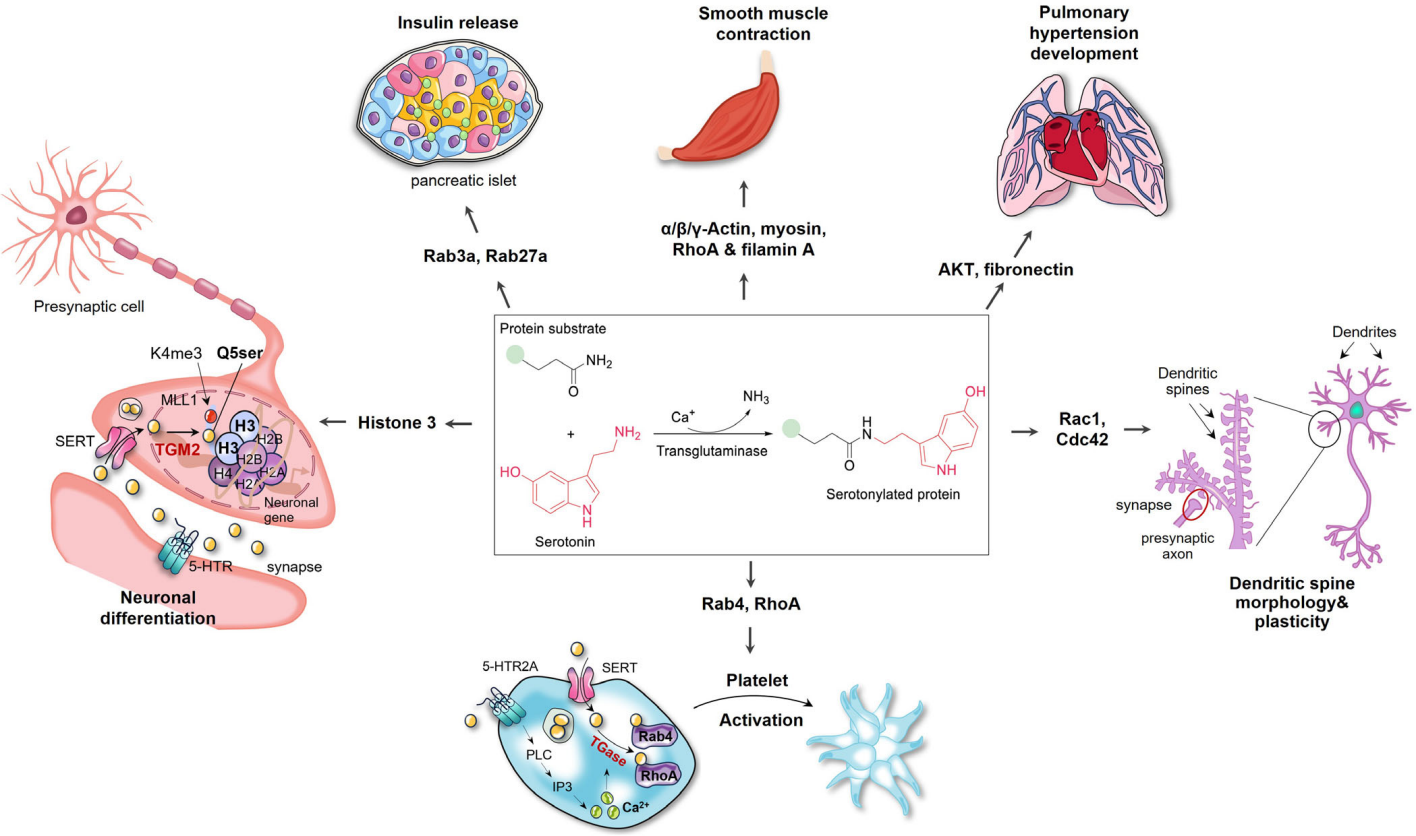
Overview of the known targets for serotonylation. Serotonylation plays a crucial role in regulating various physiological and pathophysiological processes by targeting a range of proteins, including small GTPases (Rab3a, Rab27a, Rab4, RhoA, Rac1 and Cdc42), cytoskeletal proteins (α‐actin, β‐actin, γ‐actin, myosin and filamin A), fibronectin and AKT family proteins. Serotonylation of the glutamine 5 residue on histone H3 (H3Q5Ser) represents a permissive posttranslational modification that coexists with adjacent lysine 4 trimethylation (H3Kme3), which is associated with active gene transcription. This figure was created using Adobe Illustrator and PowerPoint. AKT, protein kinase B; Cdc42, cell division cycle protein 42; IP3, inositol triphosphate; MLL1, mixed lineage leukaemia 1; PLC, phospholipase C; Rab, ras‐related proteins in brain; Rac1, ras‐related C3 botulinum toxin substrate 1; RhoA, ras homologue gene family member A; SERT, serotonin reuptake transporter; TGM2, transglutaminase 2.

One of the most striking findings on serotonylation is its remarkable function as an epigenetic marker regulating gene expression: adding serotonin molecule to the glutamine 5 residue on histone H3, known as H3Q5Ser, has been recognised as a permissive post‐translational modification which exists in conjunction with neighboring lysine 4 trimethylation (H3Kme3).^42^ H3Q5Ser potentiates the function of H3K4me3 either via stabilising H3K4me3, preventing dynamic turnover, or by improving its recognition by downstream effectors^43^ (Figure 3). Despite a smattering of identified examples of serotonylation so far, it has yielded innovative mechanistic insights and opened therapeutic avenues for a myriad of physiological and pathophysiological processes.

## 4 SEROTONIN'S PRO‐TUMOURIGENIC ROLES IN CANCER CELLS

### 4.1 Mitogenic activity through serotonin receptor signalling

Elevated 5‐HT, as well as its receptors, has been demonstrated to be involved in oncogenic progression, as an potent trophic, mitogenic and anti‐apoptotic factor.^32^ 5‐HTRs are present in numerous types of cancer, including colorectal cancer (CRC),^19^ hepatocellular carcinoma (HCC),^44^ gastric cancer (GC),^45^ breast cancer (BC),^46^, ^47^ melanoma,^48^, ^49^ pancreatic cancer,^50^ prostate cancer (PCa),^51^, ^52^, ^53^ lung adenocarcinoma,^54^ ovarian cancer (OC),^55^ bladder cancer^56^ and cholangiocarcinoma^57^ (Table 1). The intracellular reaction to 5‐HT varies between normal colon cells and CRC cells, since 5‐HT facilitated CRC cells growth without increasing the cell division rate of normal colonic crypt cells.^58^ The growth of normal colonic crypts is regulated by endocrine and autonomic neural mechanisms, while the division of CRC cells only requires endocrine signalling.^59^ 5‐HT stimulates the growth but inhibits apoptosis of CRC cells through a variety of 5‐HTRs.^60^, ^61^, ^62^, ^63^, ^64^ HCC cells express different serotonin receptors, which were demonstrated to increase tumour cell proliferation and metastasis.^44^, ^65^ 5‐HT also inhibited autophagy through HTR2B in HCC, leading to continuous phosphorylation of p70s6k and 4E‐BP1, two downstream targets of mammalian target of rapamycin (mTOR).^66^ In a cultured murine melanoma cell line, zebrafish embryos and human skin, the protein kinase A/cAMP‐response element binding protein (PKA/CREB) signalling pathway has been revealed as a mediator of HTR2A, promoting melanogenesis.^67^

**TABLE 1:** The expression of 5‐hydroxytryptamine (5‐HT) receptors in different cancer.

Cancer type	5‐HTRs expression	Experimental models	Reference
Colorectal cancer	1A, 1B, 1D, 1F, 3	Xenograft tumour model of human colorectal cancer cell lines (LoVo, HT29, SW480) and mice colorectal cancer cell (CT26, MC38) Orthotopic metastatic mouse model (HCT116) Tumour organoids isolated from human colon cancer tissues AOM/DSS‐induced colorectal cancer mouse model	^19^, ^60^, ^61^, ^62^, ^63^, ^64^, ^118^, ^161^, ^162^, ^164^
Hepatocellular carcinoma	1B, 2B	Human hepatocellular cancer cell lines (Huh7 and HepG2)	^44^, ^65^, ^66^, ^83^, ^84^
Gastric cancer	2A, 2B, 3A, 7	Human stomach cancer tissues Human gastric cancer cell lines (AGS and HGC27)	^45^, ^86^
Breast cancer	1A, 1B, 1D, 2A, 2B, 2C, 3, 4, 7	TNBC cell lines TNBC xenograft models established using LM2 and MCF10 Ca1a cell lines Tissue microarrays Hormone‐responsive breast cancer cell lines (MCF‐7, T47D)	^46^, ^47^, ^68^, ^69^, ^70^, ^71^, ^82^, ^85^
Melanoma	2A, 2B	Murine melanoma cell line (B16F10) Human melanoma cell lines (SK‐MEL‐2) Human skin and zebrafish embryos Primary tumours of various patients diagnosed with uveal melanoma cell lines	^48^, ^49^, ^67^
Pancreatic cancer	1B, 1D, 2B	Human pancreatic cancer cell lines (AsPC‐1, BxPC‐3, Capan‐2, CFPAC‐1, HPAC, PANC‐1 and SW1990) Tissue microarrays Patient‐derived xenograft mode	^50^, ^72^, ^73^, ^74^
Prostate cancer	1A, 1B, 1D, 2B, 4	PC cell lines (PC‐3, DU145, LNCaP) Benign prostatic stromal cell line (human prostate cell preparation) Xenografts of PC‐3 cells	^51^, ^52^, ^53^, ^76^
Lung cancer	1A, 1B, 7	Chronic stress and depressive‐like behaviour murine model Human lung adenocarcinoma patients tissue samples Lung small cell carcinoma cell line GLC‐8	^54^, ^77^, ^78^, ^79^, ^80^
Ovarian cancer	1A, 1B, 2B, 4	Human ovarian cancer tissues	^55^
Bladder cancer	1A, 1B	Human bladder cancer cell lines (HT1376) Human bladder cancer tissue specimens	^56^

Abbreviation: AOM, azoxymethane; DSS, dextran sulfate sodium salt; TNBC, triple‐negative breast cancer.

Mammary epithelial homeostatic mechanisms play a vital role in maintaining normal tissue function amidst the significant alterations linked to pregnancy, lactation and involution. As a crucial local controller of epithelial homeostasis in the breast, it has been observed that the biosynthetic capacity of 5‐HT was increased, associated with multiple alterations in 5‐HTRs expression in BC.^46^, ^68^, ^69^ These abnormal signals favour malignant progression of human BC cells.^46^, ^69^, ^70^, ^71^ 5‐HT was reported to amplify Warburg effect of pancreatic cancer cells through the PI3K/mTOR axis mediated by HTR2B‐LYN‐p85 complex.^50^ It also implicates in maintenance of cancer stem cells (CSCs) populations and gemcitabine resistance in pancreatic ductal adenocarcinoma (PDAC) through 5‐HTR7‐PI3K/AKT and 5‐HTR7/JAK2/STAT3 pathways.^72^ The downregulation of diverse 5‐HTRs in pancreatic cancer cells was revealed to suppress tumour proliferation and migration.^73^, ^74^, ^75^ In the case of PCa, 5‐HT generated by neuroendocrine (NE) cells exerted a pro‐proliferative effect on PCa cells via HTR1A‐MAPK/ERK and HTR1A‐PI3K/AKT signalling pathways and suppressed apoptosis via the HTR1B‐PI3K/AKT axis.^76^ The mitogenic effects of 5‐HT on small cell lung cancer (SCLC), involving HTR1A and HTR1D, were discovered in the 1990s.^77^, ^78^ Later, various 5‐HTR subtypes, including HTR3A, HTR3C and HTR7, were reported to promote lung adenocarcinoma proliferation^79^, ^80^ and correlate with poorer survival outcomes in lung cancer patients.^81^

**FIGURE 4:**
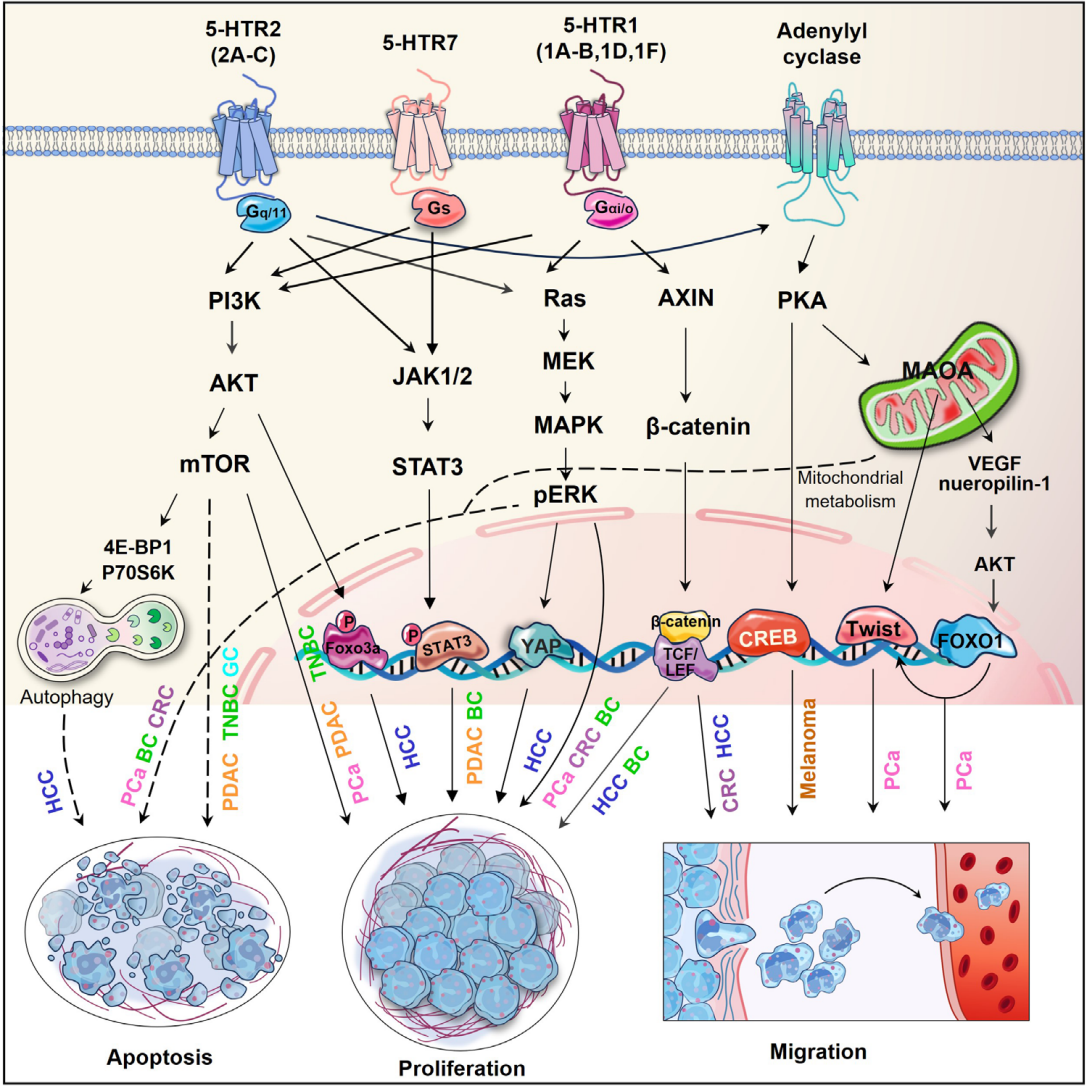
Overview of the pro‐tumourigenic effects of serotonergic signalling. Serotonergic signalling promotes tumour progression by stimulating proliferation, migration and inhibiting apoptosis in cancer cells through both canonical and non‐canonical signalling pathways. Solid black arrows indicate stimulation and dashed black arrows indicate inhibition. This figure was created using PowerPoint. AXIN, axis inhibition protein; BC, breast cancer; CRC, colorectal cancer; Foxo3a, forkhead box protein O3; FOXO1, forkhead box protein O1; GC, gastric carcinoma; HCC, hepatocellular carcinoma; LEF, lymphoid enhancer binding factor; MAOA, monoamine oxidase A; PCa, prostate cancer; PDAC, pancreatic ductal adenocarcinoma; TCF, transcription factor T‐cell factor; TNBC, triple‐negative breast cancer; VEGF, vascular endothelial growth factor.

Besides the canonical signalling pathways initiating phosphorylation cascade in the four major protein kinase pathways, several non‐canonical signalling mechanisms of 5‐HTRs have also been implicated in the pro‐carcinogenic effects of serotonin. For example, 5‐HT triggered the association between HTR1(B/D/F) and AXIN1, activating the Wnt/β‐catenin signalling. This activation occurs by preventing β‐catenin degradation, leading to promoted self‐renewal of colorectal CSCs and tumourigenesis.^19^ HTR1A interacts with TRIM21 and PSMD7 to prevent the degradation of T*β*RII mediated by ubiquitin‒proteasome. This action simultaneously suppressed the downstream Smad and MEK‐ERK‐Myc pathway, consequently hindering cytoskeletal rearrangement and epithelial‒mesenchymal transition (EMT) in BC.^82^ 5‐HT enhanced BC cell proliferation by PKM2‐facilitated glycolysis, a process dependent on HTR2A/JAK1/STAT3 signalling.^71^ HTR1D stabilised PI3KR1, exerting a potent oncogenic effect on HCC, and activated the PI3K/AKT/FoxO6 pathway.^83^ 5‐HTR7 was reported to promote the growth and migration of HCC through activating Wnt/β‐catenin signalling,^84^ and support triple negative BC cell proliferation through FOXM1, and cyclin D1 signalling.^85^ In GC, HTR2B was revealed to enhance the PI3K‐AKT‐mTOR pathway independent of its interaction with receptor tyrosine kinases, instead through crosstalk with Fyn. This activation increased the expression of HIF1α and ABCD1, concurrently reducing ferroptosis^86^ (Figure 4).

### 4.2 Pro‐tumourigenesis through serotonylation

Dysregulated serotonylation has been implicated in tumourigenesis. The small G family proteins are the primary cellular targets of TGM2‐mediated serotonylation. RhoA (Ras homologue gene family, member A) was activated by TGM2‐mediated serotonylation in CRC, enhancing yes‐associated protein (YAP) expression and promoting the carcinogenesis of CRC.^87^ Serotonylation of Rac1 in PDAC was revealed to promote its activation and be essential for the trans‐differentiation process of acinar cells into acinar‐to‐ductal metaplasia (ADM), a critical determinant in PDAC development. This phenotype was leveraged to explore the administration of SSRIs as a potential intervention to prevent the development of ADM lesions in PDAC.^88^, ^89^ In addition to the small G family proteins, 5‐HT has been reported to activate mTOR1 through serotonylation and promote CRC proliferation, independent of 5‐HTRs.^90^

### 4.3 Other pathways

5‐HT has been shown to facilitate the progression of PDAC from chronic pancreatitis (CP) by activating RhoA/Rho‐associated, coiled‐coil containing protein kinase (ROCK) signalling cascades. The 5‐HT‐RhoA/ROCK axis subsequently increased the nuclear translocation of nuclear factor‐kappa B (NF‐κB) and the expression of α‐smooth muscle actin (α‐SMA), enhancing inflammatory reactions and fibrosis in pancreatic tissues.^91^ Intriguingly, a recent study has demonstrated that 5‐HT suppresses ferroptosis (a type of regulated cell death characterised by lipid reactive oxygen species accumulation and iron dependency) independent of 5‐HTRs. Instead, it acts as a potent radical‐trapping antioxidant, eliminating lipid peroxidation.^92^ An in vitro study revealed that treating non‐small cell lung cancer (NSCLC) cells with 5‐HT enhanced their proliferation and migration. This effect was accompanied by the inhibition of c‐Myc ubiquitination and upregulation of SERT, thereby establishing a 5‐HT‐Myc‐SERT‐5‐HT feedback loop.^79^ SERT was also reported to be responsible for transporting serotonin into clone cancer cells, activating the RhoA/ROCK/YAP signalling and promoting carcinogenesis.^87^ Furthermore, the suppression of 5‐HT uptake by Azaphen dihydrochloride monohydrate reduced the tumourigenicity and inhibited the distant metastasis of NSCLC cells in vivo, underscoring the importance of SERT in tumourigenesis.

## 5 SEROTONIN'S ANGIOGENIC EFFECTS ON TUMOUR VASCULATURE

Serotonin also functions as an angiokine in tumour angiogenesis. Platelet activation results in a substantial release of 5‐HT in the tumour microenvironment (TME), where it can directly contact neighboring endothelial cells and activate angiogenic pathways. 5‐HT triggered a comparable array of signalling kinases as those stimulated by vascular endothelial growth factor from endothelial cells, such as PI3K‐AKT‐mTOR signalling, and the orphan nuclear receptor and transcription factor TR3.^10^, ^93^ Utilising a serotonin deficiency genetic mouse model (*Tph1^−/−^*), it has been reported that 5‐HT regulates angiogenesis in subcutaneous CRC allografts. This regulation is achieved by modulating MMP‐12 in tumour‐infiltrating macrophages, which in turn affects the generation of circulating angiostatin.^94^ Allografts of SCLC and melanoma in *Sert^−/−^* mice also displayed tumour retardation, which may attribute to decreased endothelial nitric oxide synthase expression and insufficient blood supply.^95^ The effect of serotonin on tumour vasculature is intricate, relying on its engagement with a diverse array of receptors, and presenting an opportunity to target these receptors as potential strategies to hinder tumour progression.^95^, ^96^, ^97^

## 6 IMMUNOMODULATORY FUNCTION OF SEROTONIN IN TUMOUR IMMUNITY

### 6.1 Serotonin signalling in immune cells

Immune cells express serotonergic components, including 5‐HTRs, SERT, TPH and MAO, which govern their effector capabilities and regulatory mechanisms.^98^ For example, serotonin skewed human macrophages towards anti‐inflammatory M2 phenotype via HTR2B/HTR7 and promoted a pro‐fibrotic gene signature through HTR7‐PKA axis.^99^, ^100^, ^101^ In dextran sulfate sodium salt (DSS)‐induced colitis mice, 5‐HT demonstrated anti‐inflammatory effects on macrophages through the HTR2A/NF‐κB pathway. Serotonin modulated cytokine production (such as interleukin [IL]1‐β, IL‐6, IL12 and tumour necrosis factor alpha [TNF‐α]) in monocytes by activating 5‐HTR3, 4 and 7 subtypes.^102^ It promoted the differentiation of immature CD1a^+^ human monocyte‐derived dendritic cells (DCs) following TLR3 activation through HTR2B,^103^ and reduced the release of proinflammatory cytokines from mature DCs through 5‐HTR4/HTR7‐cAMP signalling.^104^ The serotonin‐induced reduction of IL12 in DCs has been shown to reduce DC‐induced interferon‐gamma (IFN‐γ^+^) Th1 polarisation and Th17 polarisation.^103^ SERT on DCs allow for the uptake of serotonin from activated T cells and its subsequent release through Ca^2+^‐sensitive exocytosis, activating T cells.^105^ T cells possess a functional serotonergic system that enables them to produce, store, metabolise and respond to serotonin.^106^ Serotonin was reported to activate T cells through various 5‐HTR signalling pathways.^98^ It inhibits T‐cell polarisation to inflammatory Th1, or Th17 lymphocytes, whereas stimulating the proliferation and activation of anti‐inflammatory Tregs in various inflammatory settings.^107^

**FIGURE 5:**
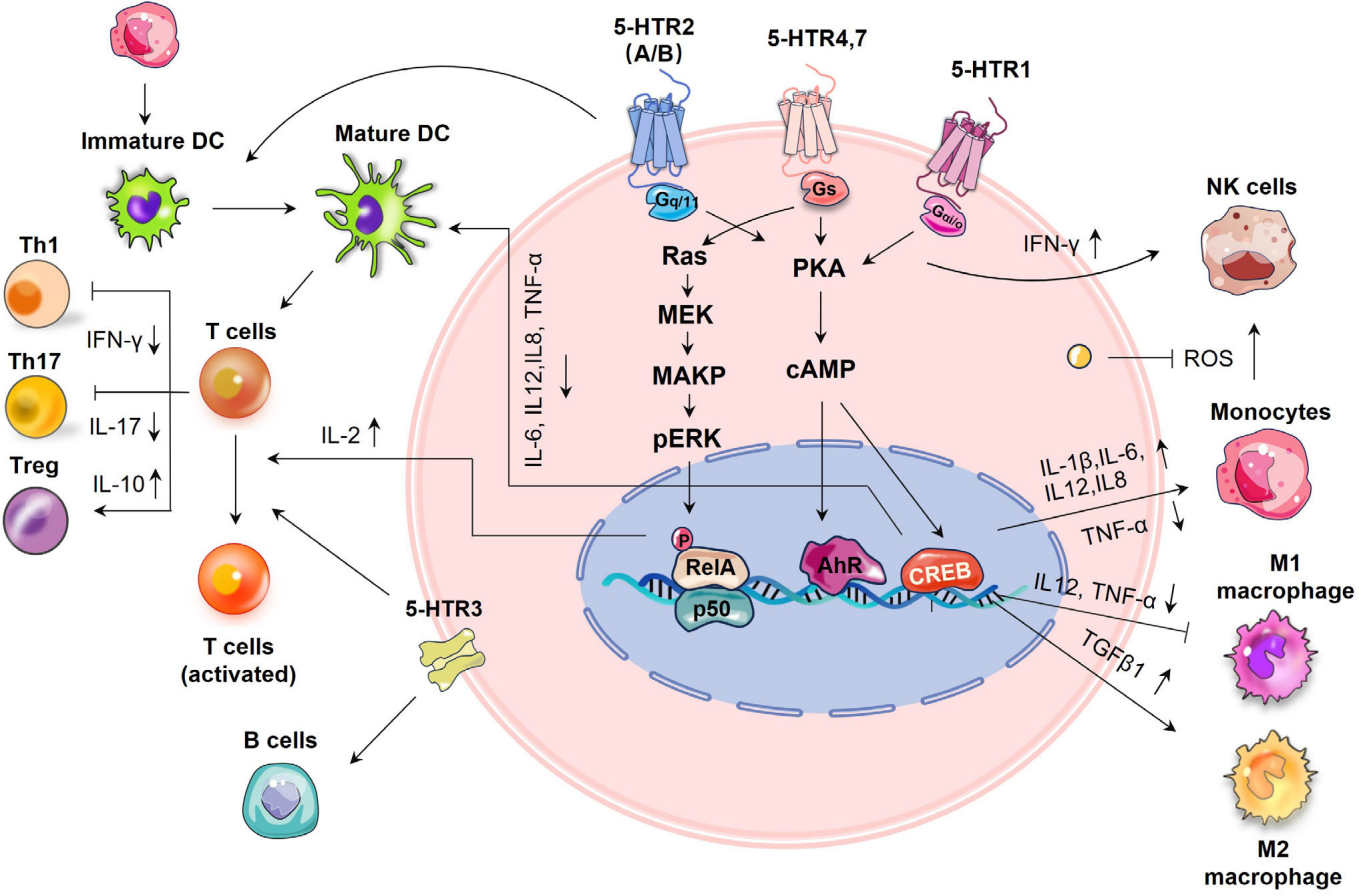
Serotonin signalling influences the immune responses. Serotonin signalling plays a key role in regulating the activation, proliferation and differentiation of T cells, promoting the maturation of dendritic cells (DCs), supporting B‐cell development, enhancing the cytotoxicity of natural killer (NK) cells, and stimulating the polarisation of macrophages towards the M2 phenotype. This figure was created using PowerPoint. AhR, aryl hydrocarbon receptor; IFN‐γ, interferon‐gamma; IL, interleukin; ROS, reactive oxygen species; Th, helper T; TGF‐β1, transforming growth factor‐beta 1; TNF‐α, tumour necrosis factor alpha; Treg, regulatory T.

Serotonin has been demonstrated to enhance mitogen‐stimulated B‐cell proliferation via 5‐HTR1A and 5‐HTR3A,^108^, ^109^, ^110^ but it induced apoptosis through SERT in Burkitt's lymphoma cells, independent of 5‐HTRs.^111^ Long‐term treatment with SSRIs has been associated with an elevation of B lymphocytes in patients, implicating an intricate relationship between serotonin signalling and the determination of cell fate across different biological contexts.^112^ With autologous monocytes, serotonin was reported to boost the cytotoxic capability of natural killer (NK) cells through HTR1A signalling.^113^ In the TME, monocytes restrict the cytotoxic effects of NK cells by releasing extracellular H_2_O_2_ and myeloperoxidase. Serotonin was revealed to protect NK cells against monocyte‐induced apoptosis in vitro by scavenging peroxidase‐derived reactive oxygen species (ROS).^114^ Conversely, inhibiting serotonin uptake with SSRIs has been shown to boost the cytosolic functions of NK cells in vitro.^115^ Platelet serotonin has also been reported to enhance the accumulation of innate immune cells, including monocytes and neutrophils at the inflammation sites.^116^ Therefore, numerous evidence suggests that serotonergic signalling affects immune cells in ways that facilitate tumour development by suppressing antitumour immunity (Figure 5).

### 6.2 Serotonin signalling in the tumour microenvironment

**FIGURE 6:**
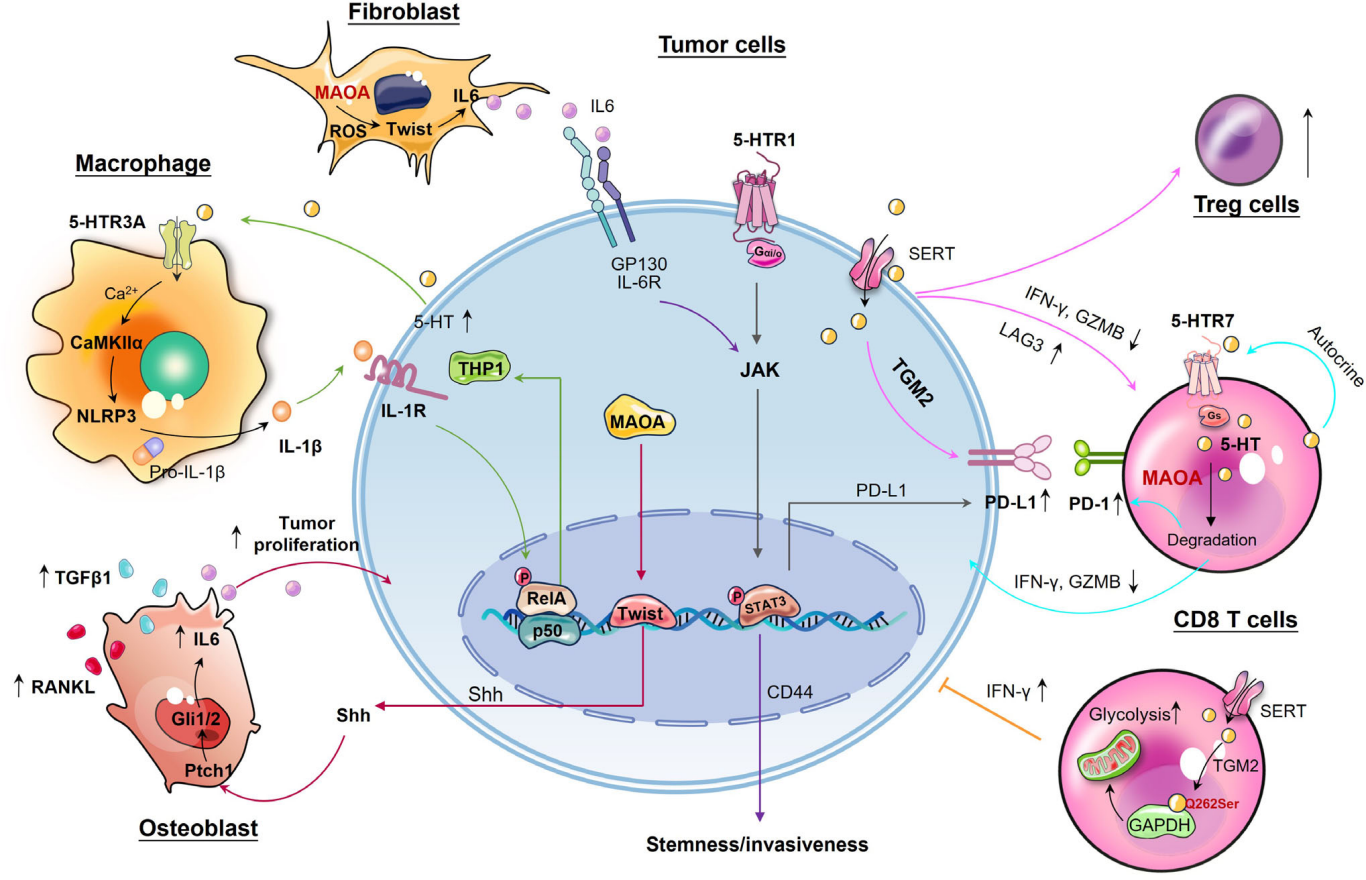
The immunomodulatory roles of serotonergic system in the tumour microenvironment (TME). The pro‐tumourigenic effects of serotonin result from the interplay between tumour cells and immune cells within the TME, leading to the suppression of CD8 T‐cell effector functions, enhanced differentiation of Treg cells and the promotion of persistent inflammation, all of which contribute to tumourigenesis. Monoamine oxidase A (MAOA) drives prostate cancer (PCa) progression through paracrine and autocrine signalling in the TME. On the contrary, serotonylation of glyceraldehyde‐3‐phosphate dehydrogenase (GAPDH) in CD8^+^ T cells was shown to enhance their antitumour activities. This figure was created using PowerPoint. CaMKIIα, calcium/calmodulin‐dependent protein kinase II alpha; GIi1/2, glioma‐associated oncogene homologue; GP130, glycoprotein 130; GZMB, granzyme B; IFN‐γ, interferon‐gamma; IL6, interleukin 6; JAK, janus kinase; NLRP3, NOD‐like receptor protein 3; PD‐1, programmed death 1; PD‐L1, programmed cell death 1 ligand 1; Ptch1, patched‐1; RANKL, receptor activator of nuclear factor kappa B ligand; ROS, reactive oxygen species; Shh, sonic hedgehog; STAT3, signal transducer and activator of transcription 3; TGF‐β1, transforming growth factor‐beta 1; TGM2, transglutaminase 2.

Besides its well‐known mitogenic roles in tumourigenesis, serotonin also functions pivotally in immune modulation within the TME. A recent investigation has indicated that 5‐HT not only influences tumour cells, but also aids in immune evasion for lung cancer patients with depression.^54^ These effects were mediated through the HTR1A/autophagy/p‐STAT3/PD‐L1 axis, which conferred resistance to cytotoxic T lymphocyte‐mediated lysis in cancer cells. Further evidence showed that peripheral serotonin could orchestrate the TME: serotonin diminished the effector capabilities of CD8^+^ T cells and upregulated the expression of PD‐L1 in subcutaneous syngeneic colorectal and pancreatic murine cancer models. Intriguingly, serotonin mediated PD‐L1 upregulation in cancer cells via serotonylation could be effectively blocked by TGM2 inhibitors.^117^ These findings underscore the importance of TGM2‐mediated serotonylation in defining the pro‐tumour effect of serotonin. The pro‐tumourigenic activities of serotonin also involve the interplay between tumour and immune cells residing within the TME: overproduced 5‐HT by CRC cells paracrinally enhanced NLRP3 inflammasome activation through HTR3A on macrophages, leading to the production of IL1β. As a result, IL1β induced *TPH1* transcription and 5‐HT synthesis in CRC cells, thereby creating a reinforcing cycle between 5‐HT and NLRP3 signalling in the TME. This loop assisted in sustaining chronic inflammation to facilitate CRC progression.^118^ Additionally, in a murine model with overexpressed human TIAM2S, ectopic TIAM2S expression provoked a pro‐inflammatory environment that facilitated CRC tumourigenesis via 5‐HT‐triggered immunomodulatory effects.^119^ SSRIs, including fluoxetine and sertraline, restored antitumour immune responses in a chronic stress‐induced mouse model of lymphoma, restricting tumour growth and cell dissemination.^120^ However, a recent study presented conflicting evidence that TGM2‐mediated serotonylation of glyceraldehyde‐3‐phosphate dehydrogenase (GAPDH) at glutamine 262 in CD8^+^ T cells induced a metabolic shift towards glycolysis, consequently promoting antitumour immune response^121^ (Figure 6).

### 6.3 Serotonin metabolism in tumour immunity

Serotonin metabolism disorders in TME can promote tumourigenesis. *Maoa* **^−/−^** mice displayed heightened T‐cell antitumour immunity and inhibited growth of colorectal and melanoma tumours in mouse synergetic models. MAOA negatively regulated the antitumour immunity of CD8^+^ T cells (including IFN‐γ, Granzyme B and PD1 expression) partly through the modulation of autocrine serotonin signalling in CD8^+^ T cells within the TME.^122^ Increased MAOA expression in PCa cells promoted bone and visceral metastasis by enhancing paracrine Shh‐IL6‐RANKL signalling in tumour‒stromal interactions. Within the bone microenvironment, MAOA stimulated osteoblast‐derived IL6 secretion and triggered skeletal colonisation via inducing osteoclastogenesis through RANKL and IL6 produced by osteoblasts, forming a feedforward loop.^123^ Reciprocally, upregulated MAOA in stromal fibroblasts was revealed to offer growth advantages to tumour cells through paracrine IL6‐STAT3 signalling, which transcriptionally activated the expression of CSCs marker CD44 in PCa cells.^124^ These findings indicate that MAOA originating from tumour or stromal cells dictates the interaction between these two cell types, favouring the reprogramming of naïve stroma towards a tumour‐supportive phenotype (Figure 6).

## 7 SEROTONIN'S ANTITUMOUR FUNCTION

Physiological responses to serotonin, albeit to a lesser extent, also display tumour‐suppressing activities, primarily mediated through a diverse array of 5‐HTRs. These receptors, acting as tumour suppressors, often demonstrate decreased expression levels within tumours. The transcriptional silencing of HTR1B in NSLCL owing to abnormal hypermethylation of its gene promoter has been implicated in endowing with growth advantage to lung cancer cells.^125^ In OC patients, reduced expression of HTR1E in peritoneal disseminated OC cells correlated with unfavourable clinical outcomes. The researchers combined the mouse stress model with the OC orthotopic mouse model and observed that HTR1E suppressed downstream pathways activated by non‐receptor tyrosine kinases Src family kinases (SRC). Consequently, the cell growth and migration that favour OC dissemination were restrained. Utilisation of HTR1E agonist or SRC inhibitor demonstrated potential in inhibiting chronic stress‐promoted OC progression.^126^ In TNBC, HTR1A expression levels were notably reduced compared to adjacent normal tissues. HTR1A inhibited the ubiquitin‒proteasome‐dependent degradation of T*β*RII by binding to TRIM21 and PSMD7, which downregulated the canonical Smad signalling and non‐canonical MEK/ERK/c‐Myc pathway. These suppressed signalling pathways inhibited cytoskeletal rearrangement and EMT in BC.^82^

Employing mouse models with defective 5‐HT biosynthesis (*Tph1*KO and Tph1^fl/fl^Villin^Cre^), researchers revealed a protective role for 5‐HT in enhancing DNA repair during the initial phases of colitis‐dependent colorectal carcinogenesis.^127^ But in colitis‐independent tumour mouse models, 5‐HT was shown to promote CRC.^94^, ^117^ The dual role of 5‐HT in CRC progression seems to rely on the stage of the disease and the underlying pathophysiological context: 5‐HT shields colorectal stem cell niche from DNA damage, thus decreasing early carcinogenic events. However, at a late stage, it facilitates the growth of established colorectal tumours. In another colitis‐associated colorectal cancer (CAC) study (azoxymethane [AOM]/DSS‐induced model), the two side effects of 5‐HT on both the onset and progression of CAC were clarified. 5‐HT‐HTR2B inhibited CAC initiation by modulating transforming growth factor‐beta (TGF‐β)‐Smad signalling, protecting from epithelial damage and inflammation. On the contrary, 5‐HT facilitated CAC progression via non‐canonical TGF‐β signal (including PI3K‐AKT and angiogenesis) in a later stage of CAC.^128^ In a murine model of syngeneic CRC, the accumulation of 5‐HT within CD8^+^ T cells was shown to enhance their glycolytic metabolism and antitumour activity, leading to the suppression of subcutaneous tumour growth.^121^ These various effects of 5‐HT in CRC underscore the intricacy of serotonin signalling and its context‐dependent nature in cancer development.

In addition, as a crucial NE stress mediator involved in various psychiatric disorders, the neural and plasma levels of serotonin are susceptible to psychological stress. For example, reduced serum serotonin levels have been detected in murine stress and rat depression models.^126^, ^129^, ^130^ The chronic unpredictable mild stress‐induced reduction of serotonin resulted in increased OC dissemination by attenuating HTR1E‐mediated tumour suppressive signalling.^126^ While elevated levels of gut‐derived serotonin were observed in a chronic mild stress mouse model, contributing to the promotion of bone metastasis in BC.^131^ These findings suggest that stress‐induced fluctuations in serotonin levels could significantly contribute to finely tuned carcinogenesis in a psychiatric context‐dependent manner.

## 8 THERAPEUTIC POTENTIAL OF SEROTONERGIC PATHWAY IN CANCER

### 8.1 5‐HT receptor‐directed therapy

Agonists and antagonists that target 5‐HTRs are commonly utilised to elucidate the roles of serotonin in tumourigenesis and the concept that serotonin receptor‐directed pharmacotherapy has emerged. For example, 5‐HTR3 antagonists are effective and safe antiemetic agents commonly used to treat nausea and vomiting following surgery and chemotherapy.^132^ Various clinical studies have been conducted or are being underway to evaluate their effectiveness in the treatment of chemotherapy‐induced nausea and vomiting in patients with different types of cancer (Table 2). Numerous in vitro studies have demonstrated the antineoplastic effects of 5‐HTR antagonists in varieties of cancer, including CRC,^64^, ^133^, ^134^ GC,^135^, ^136^ HCC,^44^ BC,^85^, ^137^ melanoma,^48^ lung cancer,^77^ pancreatic cancer,^138^ glioblastoma,^139^ OC^96^ and placental choriocarcinoma.^140^ Different inhibitors designed based on 5‐HTR1A antagonist showed inhibitory and cytotoxic effects on PCa cell lines.^141^, ^142^ A non‐selective 5‐HTRs antagonist (methiothepin) could increase doxorubicin cytotoxicity in melanoma cells,^143^ and targeting HTR2A and HTR2C with selective serotonergic receptor ligands (SER) ameliorated tamoxifen effectiveness in ER^+^ BC cells.^144^ Vortioxetine, a potent inhibitor for 5‐HTR3A, 5‐HTR7 and SERT, induced apoptosis and autophagy in GC cells through the PI3K‐AKT pathway.^145^ These antagonists are promising modulators of immune cells as well. For instance, HTR2B antagonist (SB204741) and 5‐HTR7 antagonist (SB269970) decreased the differentiation of anti‐inflammatory M2 macrophages^99^; 5‐HTR7 antagonist (SB269970) treatment reduced the velocity of migratory‐active of DCs in mouse colon,^146^ and inhibited ERK signalling in T cells.^147^

**TABLE 2:** The preclinical and clinical studies of 5‐hydroxytryptamine receptor (5‐HTR) agonists/antagonists in cancer.

Compounds	FDA approved	Target	Cancer type	Study stage in cancer	Effects in cancer
Pimavanserin (antagonist)	Yes	5‐HT2A	TNBC	Preclinical	Induced mitochondria‐dependent intrinsic apoptosis and cause cytoprotective autophagy through the PI3K/Akt/mTOR pathway in TNBC cells in vitro^137^
			PDAC	Preclinical	Inhibited the growth of PDAC by inducing autophagy mediated apoptosis^138^
			GBM	Preclinical	Suppressed the proliferation of U87 glioblastoma cells in vitro and in vivo^139^
Risperidone (antagonist)	Yes	5‐HT2A, 5‐HT2D	GC	Preclinical	Inhibited the proliferation of KATO‐III cells by inducing ROS and apoptosis in vitro and in vivo^136^
Methiothepin (antagonist)	Yes	5‐HT2	Placental choriocarcinoma	Preclinical	Attenuated mitochondrial function and induced ER stress, reducing oxidative phosphorylation, and causing metabolic shifting^140^
			Melanoma	Preclinical	Overcome the resistance of BRAFV600E melanoma cells by enhancing the cytotoxicity of vemurafenib and trametinib on these cells leading to melanoma cells death^143^
			OC	Preclinical	Suppressed OC growth by repressing mitochondrion‐mediated metabolism and inhibited angiogenesis in vivo^96^
Dolasetron (antagonist)	Yes	5‐HT3	CRC	Preclinical	Induced apoptosis in colon cancer cells by inhibiting PUM1^134^
Palonosetron (antagonist)	Yes	5‐HT3	GC	Phase II clinical trial NCT04308837	To assess this multi‐modality approach in inducing pathological complete response; decreased rates of disease progression during neoadjuvant therapy; and increased overall, disease‐free and peritoneal disease‐free survival
			BC	Phase IV clinical trial NCT05841849	Efficacy and safety of intravenous versus oral 5‐HT3 antagonists combined with NK‐1 receptor antagonists for the prevention of CINV in BC
			HNSCC	Clinical trial NCT06102447	Efficacy and safety of Palonosetron hydrochloride capsules in preventing nausea and vomiting induced by radio chemotherapy in head and neck squamous cell carcinoma
			Brain cancer	Phase II clinical trial NCT00636805	Efficacy and tolerability of palonosetron and dexamethasone in preventing acute CINV in brain tumour patients
			CRC	Phase II clinical trial NCT00381862	To study how well giving aprepitant together with palonosetron and dexamethasone works in preventing nausea and vomiting caused by chemotherapy in patients receiving chemotherapy for metastatic CRC
Ramosetron (antagonist)	Yes	5‐HT3	BC	Phase IV clinical trial NCT05326360	To evaluate the effectiveness of additional ramosetron injection for controlling late PONV after breast surgery in high‐risk PONV patients
			OC	Phase II clinical trial NCT01012336	To evaluate if new combination (aprepitant/ramosetron/dexamethasone) may improve actual CINV control in OC patients treated with taxane/carboplatin.
			Lung cancer	Preclinical	Inhibited lung cancer cell growth and migration by inducing autophagic cell death through the ERK pathway^148^
Tropisetron (antagonist)	Yes	5‐HT3	Cervical cancer	Phase III clinical trial NCT05564286	To evaluate the antiemetic effect of adding fosaprepitant to biplet regimen of tropisetron and dexamethasone for patients with cervical cancer
			Lung cancer	Preclinical	Exerted antineoplastic effects in part through modulating inflammatory and proliferating markers^149^
			CRC	Preclinical	Alleviated the tumour progression in an AOM/DOS‐induced CRC mouse model^118^
			Lung cancer	Preclinical	^148^
Tegaserod (angonist)	Yes	5‐HT4	GC	Preclinical	Inhibited the proliferation of gastric cancer cell lines and PDX models by targeting MEK1/2^151^
			Melanoma	Preclinical	Reduced tumour growth, metastases, and induced apoptosis through PI3K/AKT/mTOR signalling^150^
Vortioxetine	Yes	5‐HTR3A, 5‐HTR7	GC	Preclinical	Induces apoptosis and autophagy of gastric cancer AGS cells via the PI3K/AKT pathway^145^
Pimavanserin tartrate (antagonist)	Yes	5‐HT2A	GBM	Preclinical	Suppressed the proliferation & migration of U87 glioblastoma cells, induced G1/S phase arrest and promoted apoptosis^139^
NAN‐190 (antagonist)	No	5‐HT1A	PCa	Preclinical	Antiproliferative on PCa cell lines^51^, ^53^
8‐OHDPAT	No	5‐HT1A	TNBC	Preclinical	Inhibited the progression of TNBC via TGF‐β canonical and non‐canonical pathways^82^
CP93129 (agonist)	No	5‐HT1B	CRC	Preclinical	Stimulated growth of HT29 cells^60^
SB224289 (antagonist)	No	5‐HT1B	CRC	Preclinical	^60^
GR127935 (antagonist)	No	5‐HT1B, 5‐HT1D	CRC	Preclinical	Inhibited CRC metastasis through targeting Axin1^64^
BRL54443 (agonist)	No	5‐HT1E, 5‐HT1F	OC	Preclinical	Suppressed tumour progression in OC mouse model^126^
SB204741 (antagonist)	No	5‐HT2B	HCC	Preclinical	Inhibited cell proliferation of Huh7 cells by reducing the expression of FOXO3a^44^, ^75^
Sb‐699551 (antagonist)	No	5‐HT5A	BC	Preclinical	Reduced the frequency of tumour sphere initiating cells PDXs^70^
Metergoline (antagonist)	No	5‐HT7	TNBC	Preclinical	Antiproliferative effects on TNBC cells^85^
BJ‐1113 (antagonist)	No	5‐HT7	TNBC	Preclinical	Exhibited antiproliferative and anti‐invasive activities against MDA‐MB‐231 cells^69^
Y25130 hydrochloride (antagonist)	No	5‐HT3	CRC	Preclinical	Antimitogenic and apoptotic effect on HT29 cells^62^

Abbreviations: AKT, protein kinase B; AOM, azoxymethane; BC, breast cancer; CINV, chemotherapy‐induced nausea and vomiting; CRC, colorectal cancer; GBM, glioblastoma; GC, gastric cancer; HCC, hepatocellular carcinoma; HNSCC, head and neck squamous cell carcinoma; mTOR, mammalian target of rapamycin; OC, ovarian cancer; PCa, prostate cancer; PDAC, pancreatic ductal adenocarcinoma; PDX, patient‐derived xenograft; PI3K, phosphoinositide 3‐kinase; PONV, postoperative nausea and vomiting; ROS, reactive oxygen species; TGF‐β, transforming growth factor‐beta; TNBC, triple negative breast cancer.

In vivo, treatment with 5‐HTR3 antagonist tropisetron alleviated tumour progression in an AOM/DSS‐induced CRC mouse model^118^ and a lung cancer mouse model.^148^ Selective antagonists of HTR5A decreased the prevalence of tumour sphere initiating cells in BC patient‐derived xenografts,^70^ and a non‐selective 5‐HTR2 antagonist methiothepin was shown to boost the anticancer efficacy of paclitaxel in OC model.^96^ A retrospective clinical study demonstrated that perioperative use of 5‐HTR3 antagonist such as palonosetron or ramosetron displays potential anticancer effects with improved recurrence‐free survival in patients following open thoracotomy for lung cancer.^149^ Subsequently, the antineoplastic activities of 5‐HTR3 antagonists were confirmed and deciphered in both in vitro studies and mouse models of lung cancer.^148^, ^149^

However, an FDA‐approved HTR4 agonist called Tegaserod, which is typically employed in treating irritable bowel syndrome, has been found to effectively induce apoptosis in both BRAF^V600E^ and BRAF^WT^ melanoma. Tegaserod inhibited PI3K‐AKT‐mTOR signalling and synergistically enhanced the effects of a standard treatment, Vemurafenib, in human melanoma cell lines.^150^ It also exerted antineoplastic effects in GC by targeting MEK1/2.^151^ In addition, corroborating the antitumour properties of HTR1E in OC and HTR1A in TNBC, selective agonists targeting these receptors have been shown to suppress tumour progression, respectively^82^, ^126^ (Table 2).

### 8.2 Targeting SERT with SSRIs

Targeting serotonin transporter to prevent the absorption of serotonin confers antitumour effects in a diverse range of cancers, and the utility of SSRIs as anticancer agents is being extensively evaluated. SSRIs are the most commonly prescribed medications for alleviating depression among the general populace and individuals with cancer experiencing depressive symptoms. An epidemiological study reported that the administration of SSRIs in patients with persistent clinical depression significantly decreased their likelihood of developing CRC.^152^ Population‐based cohort studies also revealed that there is a correlation between the utilisation of SSRIs and a potentially lower chances of developing CRC in people with a family history of this disease.^153^ A recent evaluation and meta‐analysis of published observational studies unveiled that taking SSRIs was associated with decreased likelihood of developing HCC, with a dose‐dependent tendency.^154^ SSRIs have also shown an association with a decreased risk of HCC and may lower the chances of HCC in patients with HBV in a dosage‐dependent fashion.^155^, ^156^ The capacity of SSRIs to penetrate the blood‒brain barrier makes them suitable for treating conditions such as glioblastoma or brain metastases. It has been documented that the highly brain‐penetrant SSRI fluoxetine killed GBM by inhibiting sphingomyelinase activity. When fluoxetine was combined with temozolomide (a standard treatment for GBM), at doses equivalent to those within the FDA‐approved range for patients, it resulted in tumour shrinkage and extended the survival of mice carrying GBMs from patients.^157^ Furthermore, there was a correlation between the utilisation of selective SSRIs and a reduced risk of OC or bladder cancer among patients.^158^, ^159^

Blocking SERT with citalopram reversed the pro‐tumourigenic effects of serotonin and reduced the distant metastasis of CRC in preclinical studies.^87^, ^160^ Vilazodone, a selective SSRI exerted effective antimetastatic action for CRC by targeting TRIM21 (tripartite motif 21), which ubiquitinates MST2 to deactivate YAP signalling.^161^ Multiple lines of evidence have shown that the selective SSRI sertraline exhibited antitumour effects in CRC and BC cells.^162^, ^163^, ^164^, ^165^ One well‐studied mechanism involves sertraline enhancing protein levels of p53 by neutralising the action of translational controlled tumour protein on the MDM2‐p53 axis, thereby facilitating P53‐mediated apoptosis.^166^, ^167^ SSRIs sertraline and fluoxetine were reported to prevent tumourigenesis of melanoma, BC, NSCLC and HCC by targeting multiple protein kinase signalling pathways.^168^, ^169^, ^170^ The combination of these two SSRIs with sorafenib synergistically suppressed the proliferation of HCC in vitro and a mouse model of liver damage induced by diethyl nitrosamine/carbon tetrachloride (DEN/CCL4).^171^ SSRIs are recommended as a therapeutic option for BC patients to alleviate side effects such as hot flashes induced by anti‐estrogen therapy.^172^ Treatment with SSRI paroxetine‐induced apoptosis in BC cells through MAPK‐dependent ROS generation.^173^ Sertraline (Zoloft) was revealed to restrain the viability of breast tumour‐initiating cells or prostate CSCs, and synergised with chemotherapy (docetaxel), restraining the growth of BC xenograft in mice.^174^, ^175^, ^176^ Thus, SSRIs may be regarded as potential sensitisers in cancer treatment: sertraline enhanced the sensitivity of NSCLC to erlotinib by suppressing the AMPK/mTOR signalling pathway,^177^ and attenuated TRAIL resistance in lung cancer cells through inhibiting autophagic flux via upregulatoin of death receptor 5 (DR5).^178^ It also substantially reduced the tumour progression in a mouse model implanted with high‐resistant human OC xenografts when combined with Doxil (pegylated liposomal doxorubicin), acting as a chemosensitiser.^179^ In addition, SSRI fluoxetine improved the effectiveness of chemotherapy agents such as doxorubicin, mitomycin C and paclitaxel by inhibiting multidrug resistance pumps in human xenograft mouse models^180^, ^181^ (Table 3). Of note, the human equivalent doses extrapolated from the antitumour doses of sertraline (2 mg/kg)^179^ and fluoxetine (.04 or 1 mg/kg)^180^, ^181^ employed in preclinical models were lower than the doses typically administered to human patients for antidepressant purpose. Similar to other antidepressants, SSRIs display a comparable side‐effect profile, which includes GI disturbance, fatigue or insomnia, headache and transient increased anxiety following treatment initiation.^182^ Repurposing SSRIs as antineoplastic medications at higher doses may induce severe behavioural side effects. It is also crucial to ascertain whether the combination of SSRIs with conventional anticarcinogens results in synergistic or antagonistic effects. For example, certain SSRIs (such as fluoxetine, paroxetine), which exert potent inhibitory effects on cytochrome P450 (CYP450), should be avoided when used with anticancer agents (e.g., tamoxifen) metabolised via the CYP450 system.^183^

**TABLE 3:** Summary of the antitumour effects of selective serotonin reuptake inhibitors.

Compounds	FDA approved	Cancer type	Study stage in cancer	Effects in cancer
Citalopram (Celexa)	Yes	CRC	Preclinical	Inhibited CRC tumourigenesis by targeting serotonin activated RhoA/ROCK/YAP signalling^87^
		CRC	Preclinical	Reduced tumour size and the number of circulating tumour cells and metastases in an orthotopic mouse model of CRC^160^
Vilazodone	Yes	CRC	Preclinical	Reduced the metastasis of CRC cells via TRIM21‐MST2‐Hippo‐YAP signalling^161^
Sertraline (Zoloft)	Yes	BC	Clinical trial NCT00667121	To study levels of tamoxifen in the blood of women with breast cancer and in women at high risk of BC who are receiving tamoxifen together with venlafaxine, citalopram, escitalopram, gabapentin or sertraline: no results posted
		CRC	Preclinical	Exhibited proapoptotic activity with Bcl‐2 inhibition in HT‐29 cells^162^
		BC	Preclinical	Antiproliferative activity was aggravated in combination with mitochondrial inhibitors, and achieved through G1‐S‐cell cycle arrest^165^
		BC, CRC, melanoma	Preclinical	Increased the amount of p53 by neutralising TCTP's action on the MDM2‐P53 axis, thereby facilitating P53‐mediated apoptosis^166^, ^167^
		HCC	Preclinical	Induced apoptosis in HepG2 cells partially via activation of TNF‐MAP4K4‐JNK cascade signalling pathway^169^
		BC	Preclinical	Exerted antiproliferation activity by targeting the mTOR signalling pathway in a REDD1‐dependent manner^170^
		HCC	Preclinical	Synergised with sorafenib to inhibit the viability of HCC cells in vitro and in vivo via targeting the AKT/mTOR pathway^171^
		BC	Preclinical	Reduced the frequency and sphere‐forming ability of breast tumour‐initiating cells^174^
		BC	Preclinical	Combination with docetaxel synergistically reduced tumour cell proliferation and induced cell death in mammary tumour allografts^175^
		PCa	Preclinical	Targeted prostate cancer stem cells through activation of apoptosis and autophagy signalling by deregulating redox balance^176^
		NSCLC	Preclinical	Enhanced NSCLC sensitivity to erlotinib by inhibiting AMPK/mTOR signalling pathway^177^
		Lung cancer	Preclinical	Inhibited autophagic flux through upregulation of DR5 on TRAIL‐resistant lung cancer cells^178^
		OC	Preclinical	Reduces tumour growth and progression combination in combination with Doxil^179^
Fluoxetine (Prozac)	Yes	NSCLC	Phase II clinical trial NCT00005850	To test the efficacy of fluoxetine to improve patient's quality of life during chemotherapy: no results posted
		CRC, pancreatic	Preclinical	Augmented the effects of PD‐1 checkpoint blockade and triggered long‐term tumour control in mice subcutaneously inoculated with syngeneic colorectal and pancreatic tumours^117^
		GBM	Preclinical	Promoted tumour regression and prolonged the survival of mice harbouring patient‐derived orthotopic GBM in combination with temozolomide^157^
		BC, CRC	Preclinical	Synergistic growth inhibition of HT‐29 and MCF‐7 cells in combination with chemotherapy^163^, ^164^
		Lung cancer	Preclinical	Mediated ER stress and autophagy through the ATF4‐AKT‐mTOR signalling pathway^168^
		HCC	Preclinical	^171^
Paroxetine (Paxil)	Yes	CRC	Preclinical	^162^
		Lung cancer	Preclinical	^168^
		BC	Preclinical	Induced apoptosis in MCF7 cells through MAPK‐dependent ROS generation^173^
Vortioxetine	Yes	GC	Preclinical	Induces apoptosis and autophagy of gastric cancer AGS cells via the PI3K/AKT pathway^145^

Abbreviations: AKT, protein kinase B; BC, breast cancer; CRC, colorectal cancer; GC, gastric cancer; GBM, glioblastoma; HCC, hepatocellular carcinoma; mTOR, mammalian target of rapamycin; NSCLC, non‐small cell lung cancer; OC, ovarian cancer; PCa, prostate cancer; PD‐1, programmed death 1; ROS, reactive oxygen species; TCTP, translational controlled tumour protein; YAP, yes‐associated protein.

There are controversial data regarding their impact on cancer prognosis. In five retrospective cohort studies involving patients diagnosed with breast, prostate, lung, CRC and melanoma, the persistent use of SSRIs was correlated with reduced survival rates among cancer patients.^184^ A recent study revealed that duloxetine, a selective SSRI commonly used to treat major depression, can amplify the TGF‐α‐promoted activation of MAPK/AKT, JNK in HCC‐derived cell line, leading to an increase in cell migration. Nevertheless, this phenotype needs further validation since the current study relies on a single cell line and lacks a thorough mechanistic exploration.^185^

### 8.3 Inhibition of serotonin biosynthesis with TPH1 inhibitors

Depletion of peripheral serotonin with *Tph* ^−/−^ mice displayed reduced growth of syngeneic murine pancreatic and CRCs. Additionally, treatment with a TPH1 inhibitor (telotristat ethyl [TE]) in this study reduced tumour progression and simultaneously enhanced the effectiveness of anti‐PD1 therapy in mice.^117^ Administration of a TPH inhibitor also attenuated tumourigenesis in an AOM/DSS‐induced CRC mouse model.^118^ Studies have reported that treatment with a TPH inhibitor LP‐533401 suppressed the growth of BTIC and exhibited synergistic effects with chemotherapy (docetaxel), resulting in inhibition of BC xenograft growth in mice.^174^ 5‐HT produced by human cholangiocarcinoma cell lines was revealed to promote their growth in an autocrine manner. However, the proliferation of cholangiocarcinoma cells can be blocked by a TPH inhibitor *p*‐chlorophenylalanine (CPA) both in vitro and in vivo.^57^

Over the past few years, several specific inhibitors targeting TPH1 have been developed, and TE has received FDA approval for the treatment of diarrhoea in patients with carcinoid syndrome.^117^ More recently, TE is undergoing evaluation in clinical trials as a potential therapy for metastatic NE tumours.^186^, ^187^ Another phase II clinical trial is ongoing to explore the effectiveness of TE in combination with the first‐line chemotherapy for individuals diagnosed with advanced cholangiocarcinoma (ID: NCT03790111). Inhibition of serotonin biosynthesis thus represents a promising strategy for antitumour treatment (Table 4).

**TABLE 4:** Summary of the preclinical and clinical studies of tryptophan hydroxylase (TPH) inhibitors and monoamine oxidase inhibitors (MAOIs) in cancer.

Compounds	FDA approved	Target	Cancer type	Study stage in cancer	Effects in cancer
TE	Yes	TPH1	NETs	A retrospective, single‐arm, pre‐post physician panel‐based chart review of patients who received TE	Reduced peripheral serotonin and relieved carcinoid syndrome in NETs^184^, ^187^
			Cholangiocarcinoma	Phase II clinical trial NCT03790111	A safety and efficacy study of XERMELO + first‐line chemotherapy in patients with advanced biliary tract cancer (TELE‐ABC)
			CRC, pancreatic	Preclinical	Augmented the effects of PD‐1 checkpoint blockade and triggered long‐term tumour control in mouse syngeneic colorectal and pancreatic tumours^117^
4‐Chloro‐DL‐phenylalanine (Fenclonine)	No		CRC	Preclinical	Alleviated tumour progression in an AOM/DSS‐induced CRC mouse model^118^
LP‐533401	No		BC	Preclinical	Reduced BTIC frequency after transplanting drug‐treated tumour cells into immune‐compromised mice^174^
*p*‐Chlorophenyl alanine	No		Cholangiocarcinoma	Preclinical	Blocked the proliferation of cholangiocarcinoma cells in vitro and in vivo^57^
Clorgyline	Yes	MAOA	PCa	Preclinical	Reduced PCa xenograft growth in mice^189^
				Preclinical	Inhibited tumour cell proliferation and reduced expression of CSC markers^190^
				Preclinical	Reduces PNI in vitro and tumour‐associated neurogenesis in vivo^191^
				Preclinical	Decreased growth and proliferation of androgen‐sensitive and castration‐resistant prostate cancer cells^193^
				Preclinical	Restored enzalutamide sensitivity to suppress EnzR cell growth^194^
Phenelzine	Yes	MAOA and MAOB	PCa	Phase II clinical trial NCT02217709	Demonstrated efficacy in patients with biochemical recurrent castrate‐sensitive PCa^197^
			PCa	Phase II clinical trial NCT01253642	To study how well giving phenelzine with docetaxel works in treating patients with PCa that is growing, spreading, or getting worse after first‐line therapy with docetaxel.
			CRC, melanoma	Preclinical	Suppressed tumour growth in mouse syngeneic and human xenograft tumour models in a T‐cell‐dependent manner^122^
			PCa	Preclinical	^194^

Abbreviations: AOM, azoxymethane; BC, breast cancer; CRC, colorectal cancer; CSC, cancer stem cell; DSS, dextran sulfate sodium salt; NETs, neuroendocrine tumours; PCa, prostate cancer; PD‐1, programmed death 1; TE, telotristat ethyl.

### 8.4 Targeting MAOA for PCa therapy

The heightened expression of MAOA has been observed to exhibit a strong correlation with the Gleason grade and preoperative serum prostate‐specific antigen (PSA) levels of patients with PCa, making it a promising biomarker for PCa prognosis.^188^ MAOA‐dependent HIF1α‐VEGF‐A‐FOXO1‐TWIST1 pathway promoted PCa growth and metastasis. Knockdown or pharmacological blocking MAOA suppressed tumour growth and metastasis in PCa xenograft mouse model.^189^ In a prostate conditional *Maoa* knockout mouse model, PCa development was significantly inhibited with slowed proliferation and reduced expression of CSC markers, such as CD44, α2β1 and CD133.^190^ Additionally, MAOA was found to promote perineural invasion (PNI) of PCa, characterised by the infiltration of tumour cells into surrounding nerves, serving as a prognostic indicator for poor outcomes and survival in this disease. At the mechanistic level, MAOA triggered the activation of SEMA3C through a Twist1‐dependent transcriptional process, subsequently stimulating cMET to enhance PNI through interactions with co‐activated NRP1 and PlexinA2^191^ (Figure 4). While MAOA expression is not universally upregulated in all cancer types. For example, in GC tissues, the expression of MAOA was found to be notably reduced, which correlated with an unfavourable patient prognosis.^192^

Substantial evidence indicates that targeting MAOA blocks PCa proliferation and metastasis,^189^, ^190^, ^191^ restores enzalutamide sensitivity,^193^, ^194^ and revokes immune suppression.^122^, ^195^, ^196^ Importantly, clinically available MAOIs, which are utilised in the treatment of depression and various neurological conditions, have demonstrated promising outcomes against PCa in both experimental models and clinical studies, presenting a promising chance for their repurposing as a treatment for PCa. A phase II clinical trial (ID: NCT02217709) reported that phenelzine, a non‐selective MAOI, shows effectiveness since serum PSA levels decreased in individuals experiencing biochemically recurring, castration‐sensitive PCa.^197^ In another phase II clinical trial (ID: NCT01253642), a treatment regimen involving both phenelzine and docetaxel was administered to patients with advanced PCa. Phenelzine hinders tumour progression and potentially improves the efficacy of docetaxel. Further studies are imperative to potentially broaden the application of MAOIs in cancer therapy, facilitating their transition into clinical practice (Table 4).

## 9 CONCLUSIONS AND PERSPECTIVES

Serotonin (5‐HT) was first identified over 7 decades ago and initially characterised as a vasoconstrictor. It is a versatile neurotransmitter and peripheral hormone, with emerging mitogenic functions in carcinogenesis. However, conflicting evidence, coupled with the complex and multifaceted neoplastic effects of the serotonergic system, poses significant challenges for repurposing serotonergic‐targeted pharmaceuticals in cancer therapy. A more comprehensive understanding of the context‐dependent neuro‐immuno‐endocrine mechanisms through which the serotonergic system modulates carcinogenesis is essential for developing future evidence based and holistic therapeutic strategies.

The pro‐tumour functions of serotonin involving in proliferation, anti‐apoptosis, invasiveness and angiogenesis, were initially identified, and primarily studied in in vitro systems, largely due to its mitogenic properties. Although the carcinogenic effects of serotonin are gaining momentum in recent years, contradictory findings have emerged, especially with the development of 5‐HTR‐specific knockout and peripheral serotonin deficient (*Tph^−/−^*) mouse models, as well as 5‐HTR subtype‐selective drugs. The conflicting roles of serotonin in the pathogenesis of tumour may be attributed to the fact that (1) the tissue‐specific expression patterns of 5‐HTRs, for example, HTR1E has been observed to be downregulated in OC patients and functions as a cancer suppressor in the context of chronic stress‐promoted OC progression; (2) the dose‐dependent mitogenic effects of serotonin, for example, higher doses of 5‐HT stimulate cell growth, while lower concentrations induce vasoconstriction in tumour vessels, resulting in repressed tumour progression; (3) the stage of carcinogenesis and the underlying pathophysiological context, for example, 5‐HT facilitated DNA repair during the early phases of colitis‐dependent CRC, but promoted CRC progression in the colitis‐independent CRC through a plethora of mechanisms; (4) the complicated and obscure roles of serotonin in orchestrating the TME, for example, serotonin mediated immune evasion in lung cancer mouse model by upregulating PD‐L1 expression via serotonylation, whereas circumstantial evidence indicates that serotonin contributes to enhance NK cells’ cytotoxic capabilities. Moreover, the intricate biological responses to serotonin are shaped by the combined effects of multiple 5‐HTRs, and the dysregulated expression patterns of 5‐HTRs are often observed in the progression of certain tumours. Therefore, gaining a more profound comprehension of the underlying mechanisms of serotonin/5‐HTRs axis‐mediated alterations in carcinogenesis, exploring serotonylation and other modes of serotonin signalling, and elucidating the intricate interplay between serotonin and the TME possess great promise for the development of serotonergic‐targeted therapies against cancer.

5‐HTRs, SERT and serotonin biosynthesis/metabolism pathways are potential molecular targets in cancer‐directed pharmacotherapy. 5‐HT binding agents, SSRIs, 5‐HT synthesis inhibitors and MAOIs offer valuable clinical options for leveraging the translational potential of serotonin‐mediated tumourigenesis, given the established arsenal of these drugs.^198^ However, more extensive in vivo studies that incorporate tissue‐specific knock out strategies are urgently needed to thoroughly evaluate the therapeutic vulnerabilities, efficacy and safety profile of these medications in cancer treatment. The advancement of highly selective drugs that singularly target individual subtypes of 5‐HTRs based on their structural characteristics is essential for minimising polypharmacology and reducing the risk of side effects. It is also crucial to determine the optimal treatment regimens for different types of cancer and patient populations. With the notion of serotonergic‐targeted drugs for cancer, the therapeutic landscape is gradually unfolded. Elucidating the clinical benefit and improving tailored therapeutic approaches with these drugs will necessitate evidence based and scientifically guided clinical trial designs and comprehensive endeavour to discover predictive biomarkers.

## AUTHOR CONTRIBUTIONS

*Writing, figure preparation, conceptualisation and critical revision*: Mei Song and Weiling He. *Searching literature and initial draft preparation*: Lulu Chen and Shuting Huang. *Editing*: Xiaoxue Wu. All authors have read and agreed to the final manuscript.

## CONFLICT OF INTEREST STATEMENT

The authors declare they have no conflicts of interest.

## ETHICS STATEMENT

Not applicable.
